# “It is unbearable to breathe here”: air quality, open incineration, and misinformation in Blantyre, Malawi

**DOI:** 10.3389/fpubh.2023.1242726

**Published:** 2023-10-12

**Authors:** Elizabeth Tilley, Hope Chilunga, Jonathan Kwangulero, Lars Schöbitz, Saloni Vijay, Heiko Heilgendorff, Marc Kalina

**Affiliations:** ^1^ETH Zurich, Department of Mechanical and Process Engineering, Global Health Engineering, Zurich, Switzerland; ^2^Department of Environmental Health, Malawi University of Business and Applied Sciences, Blantyre, Malawi; ^3^School of Mathematics, Statistics and Computer Science, University of KwaZulu-Natal, Durban, South Africa; ^4^School of Engineering, University of KwaZulu-Natal, Durban, South Africa

**Keywords:** waste management, healthcare waste, trash burning, air quality, Malawi, urbanisation

## Abstract

Blantyre, Malawi’s Queen Elizabeth Central Hospital (QECH), or Queen’s, as it’s known locally, is the country’s largest public hospital. However, Queen’s is not served by regular municipal waste collection. Rather, most hospital waste (infectious and non-infectious) is gathered by grounds staff and openly burned, in several constantly smouldering piles, sending up clouds of smoke. Speaking directly to an identified knowledge gap on air quality impacts linked to trash burning and the paucity of African urban dwellers’ voices on air quality issues, this study employed a mixed-methods approach to both quantitatively measure the air quality around QECH, and to qualitatively investigate the perceived impacts amongst staff and caregivers. Low-cost sensors measuring particulate matter (PM) with particle sizes less than 10 μm (PM_10_) and less than 2.5 μm (PM_2.5_), expressed as the mass of PM per volume of air (μg PMx/m^3^ air) were recorded every 5 min at 8 locations across the QECH for 2 months. Qualitative data collection consisted of 56 interviews with patients, caregivers and hospital staff (including janitorial and maintenance staff, nurses, doctors, and administrators). Our results show that safe air quality thresholds are consistently exceeded across space and time and that the most problematic air quality surrounds the shelter for caregivers and those receiving treatment for HIV/AIDS. Moreover, staff and visitors are severely impacted by the poor air quality within the space, but feel powerless to make changes or address complaints. Waste management interventions are desperately needed lest the patients who arrive at Queen’s leave with more health issues than the ones with which they arrived.

## 1. Introduction

Built in 1964, Malawi’s year of independence, Queen Elizabeth Central Hospital (QECH), or Queen’s, as it’s known locally, is the country’s largest public hospital. Designed in the Tropical Modernism style of architecture popular within Africa in the late colonial period, Queen’s open-plan form sprawls across a broad swath of central Blantyre, with dozens of wards, specialised facilities, and administrative buildings, linked together by a bewildering maze of covered walkways. A hive of around-the-clock activity, Queen’s bustles from dawn to dusk with a constant stream of patients, drawn from across the country, and maintains a city- like feel even after dark as staff and stay-in family members cook and care for their in-patient dependants. Yet, for the first-time visitor, it’s not the architecture or the crowds that are the most striking when entering the grounds, but the smell. Immediately noticeable, even from the road outside the hospital, Queen’s is smothered by an acrid-smelling, white smoke, which hangs over the grounds day and night: the product of several constantly burning fires spread across the campus. Breathing this air, when a significant amount of burning is occurring, can be incredibly uncomfortable. Moreover, the tropical architecture, designed to let air and light circulate, means that even indoors, within patient wards, surgery theatres, and treatment areas, the air quality can also be unbearable. Poor air quality associated with burning, through released particulate matter (PM), has been linked to multiple negative health outcomes like asthma, heart disease, heart failure, stroke, and cancer among others (1–3). In a space of healing and recovery, why is such burning occurring, and how can it continue to persist?

**Figure 1:**
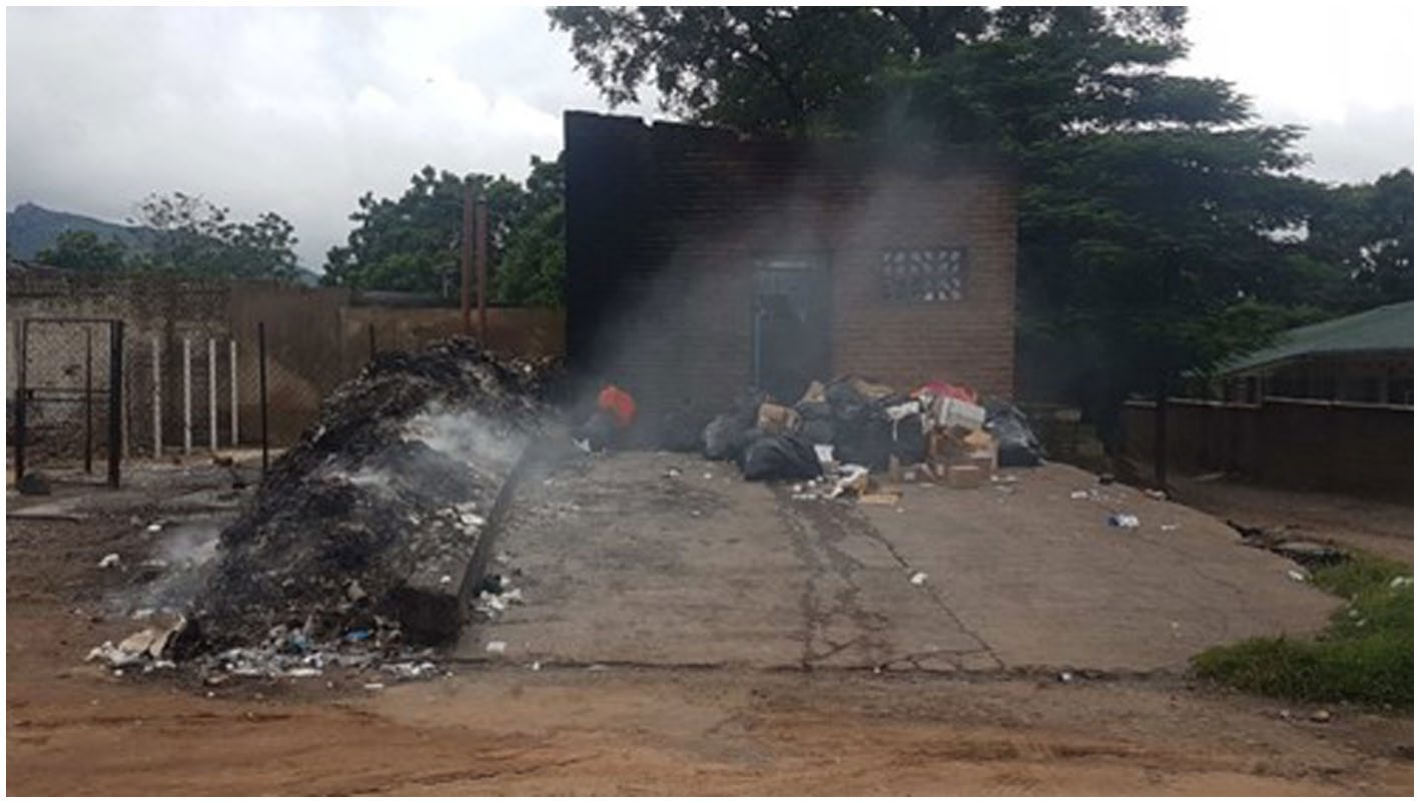
Open burning at QECH, with the old incinerator building in the background (Authors).

**Figure 2:**
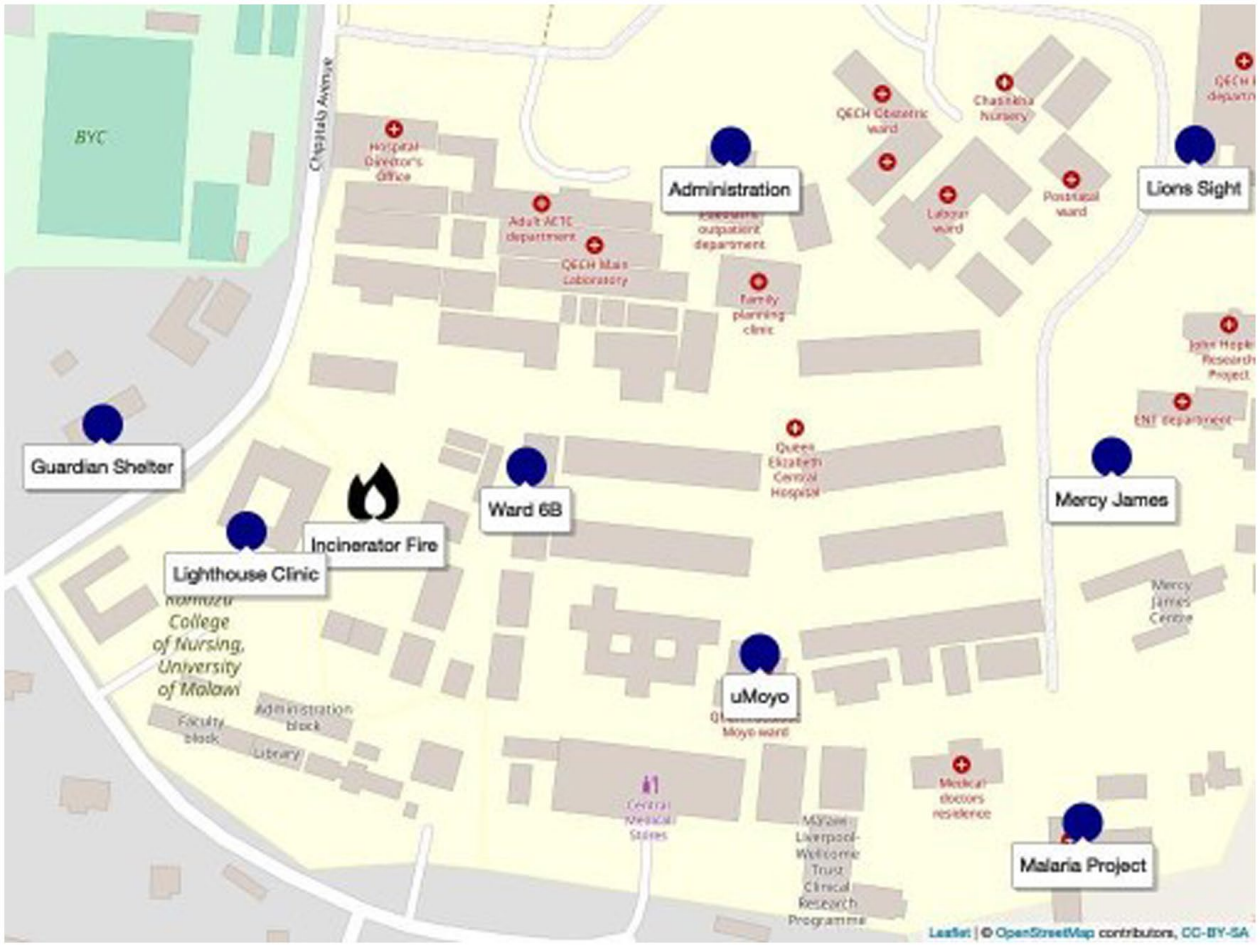
Sensor locations within QECH.

Queen’s produces an immense amount of waste that it is unable to efficiently manage. The dozens of wards, offices, and kitchens, serving the hundreds of patients and staff, generates considerable infectious medical waste, mixed domestic and office waste, and kitchen waste, which needs to be removed and disposed of daily. Queen’s is not served by regular municipal waste collection. What municipal collection that is done is *ad-hoc*, sporadic, and based on the hospital’s available financial resources. Rather, most hospital waste (infectious and non-infectious) is gathered by grounds staff and burnt at the hospital’s incinerator, located at a central point within the hospital campus^1^ (4). However, the vast majority of the waste is not incinerated, as a new incinerator provided by the Ministry of Health in late 2019, is only able to handle a small percentage of the hospital’s waste. Furthermore, the incinerator is frequently inoperable, under repair, or without power.^2^ Rather, the waste is openly burned, in a constantly smouldering pile to the side of the incinerator building, sending up the aforementioned clouds of smoke, which choke the grounds (Figure 1). Innumerable other, smaller, fires, scattered across the QECH campus (Figure 2) contribute their part, as grounds and maintenance staff habitually burn leaves and other garden refuse. Furthermore, caregivers^3^ who reside separately on hospital grounds, cook for themselves throughout the day and night using biomass (charcoal or wood), and burn their own domestic waste. As a result, air quality at the hospital is a constant source of discussion, with patients, staff, and caregivers struggling to cope, when just breathing is, as the quote^4^ used in the title of this article, “unbearable.”

Challenges with solid waste management (SWM) are not unique to Queen’s, and remain persistent globally, particularly within African nations, and the Global South more broadly (5–8). Explanations for these disparities have centred on waste generation outpacing waste management infrastructure (7), and the high costs of waste management systems, which often prove unaffordable for many low-and-middle-income countries (LMICs) (6, 9). Waste collection is an important step within SWM systems, and is a common barrier within LMIC contexts (5). Waste inequalities between high-income countries (HICs) and low-income countries (LICs) is often most pernicious and visible at the point of collection, with some HICs achieving 100% collection rates, while some African nations have rates well below 50% (10). Inequalities persist beyond collection, however. Even once waste is collected, municipalities in LMICs may burn or dump it in non-sanitary landfills due to the absence of further treatment or disposal options (11). According to Kaza et al. (12), 90% of waste in LIC is disposed of in unregulated dumps or openly burned, while the quantity of waste generation in these countries is expected to triple by 2050. As a result, open waste burning, in nations like Malawi, can be expected to become more prevalent.

Although hospital waste is among the common types of solid waste openly burned in LICs (in addition to municipal solid waste, sewage sludge, market or commercial waste, agricultural residues), the open burning of hospital waste has not garnered as much discussion as the burning of other waste fractions (11). The World Health Organization (WHO) defines hospital, or health-care waste, as waste generated by health-care activities, of which approximately 85% is general, non-hazardous waste, but the remaining 15% can be considered hazardous, and may be toxic, infectious, or radioactive. These infectious fractions are commonly incinerated in both high and middle-income countries, however in the absence of capacity, or costly incineration or autoclave infrastructure, open burning is often a last resort option for disposal in LIC contexts (13). The open burning of biomedical waste may reduce infection risk from potentially harmful pathogens, however, such waste may contain sharps, radioactive waste, mercury-containing instruments, and plastics, the open burning of which may result in the emission of dioxins, furans, and toxic particulate matter (11, 14). Moreover, although the sustainable and circular management of healthcare waste was highlighted at the past COP27 Climate Change Conference, the concepts, as Syms et al. (15) note, have not yet been widely taken up by healthcare systems, especially within the Global South.

Poor air quality in in African cities can be attributed to multiple factors, including vehicular emissions, dust, or the use of low-quality fuels for cooking, lighting, and heating; however, the open burning of waste has been identified as one of the key contributors to air pollution within urban areas (16–18). Although open trash burning is generally a problem in LICs, Wiedinmyer et al. (19) estimate that open trash burning contributes to 29% of the total global PM_2.5_ anthropogenic emissions. Furthermore, it is of particular concern in Sub-Saharan African (SSA) cities, which are home to 19 of the world’s 50 biggest dumpsites (20). Particulate matter, is not specific to trash burning, but rather the product of any type of incomplete combustion and is one of the most commonly measured indicators for air quality. PM is measured in terms of its particle size and generally classified into PM with particle sizes less than 10 μm (PM_10_) and those with sizes less than 2.5 μm (PM_2.5_), while one way of measuring the severity of air pollution is by expressing the mass of PM per volume of air (μg PMx/m^3^ air). PM has been linked to multiple negative health outcomes. Amongst particulate air pollution, PM_10_ and PM_2.5_ are of particular concern as they can penetrate deep into the lungs, and their exposure has been associated with asthma, heart disease, heart failure, stroke, and cancer (21, 22). In addition, burning can produce a wide range of atmospheric pollutants including short lived climate pollutants (SLCPs) such as black carbon (BC). BC emissions are a major source of fine particulate matter, with a climate change impact up to 5,000 greater than CO_2_, and significant environmental health risk. As a result, the health impacts of ambient air particle pollution may be significant, especially for the most vulnerable. For instance, the IHME (23) estimates that PM related air pollution resulted in 4.14 million deaths worldwide (in 2019), of which, 394,000 occurred in Africa. Yet, despite its importance, the story of air quality in Africa is worryingly incomplete. For instance, the WHO only collects data from 10 African countries (covering 39 cities) (24). Likewise, robust, epidemiological data from and for the African context is also lacking, though emerging data suggests strong correlations between PM and poor health outcomes (25, 26). Moving forward, it is not just the air at Queen’s which lacks clarity.

Utilising a mixed-methods approach, including a network of custom-designed air quality monitors and qualitative fieldwork with hospital staff, patients, and caregivers, the purpose of this investigation was to gain a multi-dimensional understanding of the impacts of the open burning at QECH. Specifically, this work aimed to intensively and longitudinally measure the air quality, in terms of particulate matter, at multiple locations at, and surrounding, the central burning point. Furthermore, we also aimed to qualitatively understand how affected individuals perceive air quality at QECH and understand potential health impacts.

This research responds to a several specific gaps within the body of academic air quality literature: although there has been ample discussion of air quality challenges in African cities (27–30), there is a paucity of data on air quality impacts linked to trash burning. In particular, within African cities, there has been a total dearth of scholarship on the burning of medical waste, or on air quality within hospital contexts. Furthermore, while the voices of the citizens of Northern cities (31–33) have been well documented on localised air quality issues, the voices of African urban dwellers have not been given the same consideration, and there remains a pressing need to understand how these populations experience the impacts of the open burning of trash within their communities and understand potential risks.

Findings suggest that particulate matter concentrations are routinely above the WHO guidelines and are especially worrisome at several locations. Over the course of the 2 month study, the hazardous limits for both PM_10_ and PM_2.5_ were exceeded at all locations except for the Lions Sight hospital. The limits were exceeded fewer than 50 times at five of the locations, but the monitors at the Lighthouse Clinic and the Guardian Shelter recorded more than 50 instances above the hazardous limits. The extremely hazardous air quality at the Guardian shelter is mostly from cooking over open fires, while the PM at the Lighthouse Clinic is a function of being located directly adjacent to the smouldering waste pile.

Interviews show that patients, staff, and caregivers alike, are keenly aware that the air quality at Queen’s is poor, with most respondents reporting frequent respiratory-related illness. Many also linked the smoke to potential long-term health complications and expressed a belief that the pollution could be contributing to other, more potentially life-threatening diseases, such as asthma, cancer, and tuberculosis. Moreover, the tropical design of hospital buildings has rendered most coping mechanisms ineffective, with staff only finding relief at home: relief that is not available to the hundreds of patients and caregivers who sleep at QECH or are unable to leave.

In many contexts, waste burning is a necessary, but sporadic event with limited exposure. However, in the context of a large hospital, serving the sickest and poorest, this study shows that the consistent, and highly toxic smoke produced is an unrelenting and unnecessary burden that must be addressed, lest the patients leave in worse condition than in which they arrived.

## 2. Materials and methods

This study utilised a mixed-methods approach to both quantitatively measure the air quality at eight locations around QECH, and to qualitatively investigate the perceived impacts amongst staff and caregivers. Although the work was conducted over one sustained period in late 2019, it must be contextualised within 5 years’ experience of research and activism within QECH by the authors.

### 2.1. Study site

The sensors were located within the QECH campus in consultation with hospital management. The goal was to locate sensors across the campus with a range of distances and directions from the incinerator, though the final decision was based on accessibility, the permission of the unit’s head, and convenience. Each of the locations is briefly described below and shown in Figure 2:

- **Lions Sight First Eye Hospital**, often shortened to **Lions Sight**, is the largest eye hospital in Malawi and serves as the main teaching eye hospital for the Kamuzu University of Health Sciences (formerly College of Medicine). It is staffed by 5 consultants and a team of clinical officers and nurses. It provides a mix of public (no cost) and private (at cost) services.
- **The Blantyre Malaria Project (BMP)**, established by Professors Terrie Taylor and Malcolm Molyneux, has carried out clinical research and patient care in the area of paediatric malaria, specifically cerebral malaria, for more than 30 years^5^. Its research infrastructure includes an administrative team, an inpatient research ward, an MRI centre, and an outpatient research clinic in Ndirande township and in other districts outside of Blantyre.
- **Ward 6B** is a male ward for trauma and orthopaedic patients. It was established as part of the original hospital design. It is a 60-bed ward with a dedicated nursing station and treatment rooms.
- **The Blantyre Lighthouse Trust Clinic**, also known as **Umodzi Family Centre** or simply, the **Lighthouse Clinic**, is one of four operating across Malawi. The clinics work in collaboration with the Ministry of Health (MOH) to provide integrated HIV testing, treatment and care for people living with HIV.
- The **Moyo Nutritional Rehabilitation and Research Unit**, known on campus as **uMoyo**, is a 57-bed nutritional rehabilitation unit (NRU) for treating infants and children with severe malnutrition and acute illnesses. It is one of 104 operational NRUs in Malawi. It also has an outpatient therapeutic feeding program (OTP) for children with malnutrition who can be treated outside of the hospital.
- **The Mercy James Institute for Pediatric Surgery and Intensive Care (MJC)**, shortened on campus to **Mercy James**, was opened in 2017 by the NGO Raising Malawi, which was founded by Madonna. With 3 operating rooms and 50 beds, it is the first and only first dedicated paediatric surgery hospital in Malawi. Prior to its opening, QECH had fewer than 10 intensive care beds.
- The **Administration Building** is the operational hub of the campus, containing mostly offices and meeting rooms.
- When a patient arrives at QECH, she or he will usually arrive with a guardian: someone to cook for them, buy medicine, do their laundry, and help them bathe. The **Guardian Shelter** is a gender-separated concrete floor shelter for sleeping along with a cooking pavilion and toilet/shower block that are maintained by a local NGO, Chira fund. Though simple, the facility is secure and offers one of the only free/accessible toilet facilities for visitors on the QECH campus.

### 2.2. Waste management

As the largest public hospital in the country, Queen’s produces an immense amount of waste. Hospital staff, such as doctors and nurses, primarily generate and handle medical waste (dry, wet, sharps), which is disposed of in separate waste bins labelled accordingly within the ward. Hospital visitors (caretakers and patients) generate food waste and are advised by staff to discard such waste in waste bins outside. Cleaning staff are responsible for emptying bins and maintaining the cleanliness of indoor and outdoor spaces. Collected bags of waste are temporarily stored in the sluice room, and the night-shift cleaners are responsible for transporting the waste on wheelchairs or trolleys to the incinerator for burning.

Yet, according to respondents and our own observations, waste management procedures within the hospital are fraught with challenges which impact the implementation of best practices. These challenges include the lack of a coherent hospital-wide waste management policy, insufficient waste management training for staff, and a persistent lack of material and financial resources. For instance, although a representative from the hospital administration was able to articulate a set of standard operating principles (SOPs) for waste management, doctors and nurses were generally limited in their understanding about its contents. Furthermore, cleaners, who are responsible for day-to-day cleansing and waste disposal, yet rate at the bottom of the institutional hierarchy at Queens, were unable to access or analyse hospital policy, and generally tended to rely on, what one cleaner^6^ described as, “experience” and “human judgement” when handling potentially hazardous waste. Moreover, although nearly all doctors and nurses had prior education on waste management best practices, cleaners began their waste handling duties without any prior waste management experience, and although there is an orientation programme for new hires, there is no regular refresher training or capacity building regarding waste for existing staff, at any level.

Despite its national prominence and international reputation for academic medical research, Queen’s is plagued by constant shortages of human and material resources. Yet, these shortages are not shared equally across the space, with a sharp division in cleanliness, resources, and quality of care between public wards, supported by the Malawi Ministry of Health, and the private wads with international funding, such as the aforementioned Mercy James Institute. For staff, these shortages have manifested in poor salaries, missed pay cheques, failing infrastructure and limited supplies (4). Chronic shortages of black bags, waste containers and cleaning staff which has resulted in limited waste segregation, i.e., highly infectious items (bandages, gloves, syringes, and other sharps) are disposed of along with non-hazardous waste (food, newspapers, packaging). As a result, all waste is mixed, necessitating more burning than would be necessary if non-hazardous waste was diverted, and contributing to workplace risk, with reports of accidental exposure to potentially infected waste, including accidental pokes from improperly disposed of sharps, common amongst hospital staff (34). Regardless, separation ends at the point of collection. The hospital is not serviced by regular municipal waste collection, and as a result, nearly all of the waste produced by the hospital, hazardous and non-hazardous, has to be disposed of on-site, either within the limited capacity of the hospital incinerator, or more commonly, through open burning. The constantly smouldering pile of waste puts forth a continuous cloud of grey smoke, which mingles with the dozens of other fires on the grounds, from cooking fires and burning garden waste, and blankets Queen’s in a permanent cloud of foul smelling haze.

### 2.3. Measuring air quality

#### 2.3.1. Hardware and software

The PM monitoring device centres around a Raspberry Pi 3 Model B single-board computer complete with an operating system and storage space. The added advantage of storage space is that the data are not lost if the internet connection breaks down. Because the Pi can only read digital signals, we needed to include an analogue-to-digital (ADC) converter. The particulate matter is measured by a Nova PM SDS011 High Precision Laser sensor that measures particles at 2.5 and 10 μm in diameter in μg/m^3^. A list of the specific hardware and software components can be found in Appendix B.

#### 2.3.2. Installation

The choice of where the sensors were installed was done in collaboration with the QECH administration, but the position of the sensors at the individual buildings were decided by the research team. Four air quality sensors were installed on the outside of buildings (Guardian Shelter, Mercy James, Malaria Project, Lighthouse Clinic) while the remaining four sensors were installed inside (Ward 6B, Administration, Lions Sight, uMoyo). All of the sensors were mounted to a wall at a height of approximately 2 m from the ground with the help of QECH maintenance department personnel. This height was chosen to both capture the approximate breathing zone and to prevent the public from tampering with the equipment. Additionally, we ensured that each inhalation pipe that pulled in ambient air was freely protruding in the building (room) to capture the air quality.

Because connecting each unit to a power supply was not possible, each monitoring unit was equipped with an external battery (5,000 mAh) which powered the sensor unit for 3 days continuously. To avoid any down time, we changed the batteries every 2 days. Data were also collected from the sensor at the time of battery replacement. The sensors were capable of being connected to Wi-Fi, a feature which enabled wireless data access.

#### 2.3.3. Qualitative methods

Qualitative data collection consisted of 25 interviews with caregivers and hospital staff (including janitorial and maintenance staff, nurses, doctors, and administrators) conducted in October and November 2019, as well as an additional 31 interviews conducted with staff, patients, and caregivers spread around the hospital’s various wards and departments in January and February 2021. Interviews were semi-structured, and included questions on hospital waste management practices, perceived best practices, and workplace health, safety, and risk. The first round of interviews included specific questions on air quality, while the second round focused on waste management more broadly. However, the semi-structured nature of the interviews allowed for the participants to raise topics of interest, and for unexpected themes to emerge. These interviews were supplemented by participatory observation recorded within detailed field notes, and supported by several years of sustained involvement by the authors in the waste management practices at the hospital.

Interview respondents were chosen using a purposive or judgement sampling regimen, i.e., a subjective sampling method in which respondents are selected based on their ability to effectively contribute to the study’s research objectives (35). All respondents were purposively selected based on availability, willingness to provide written, informed consent, and their individual insight into the questions posed within the study. Interviews were conducted in the local language (Chichewa), audio recorded, and transcribed into English. Participation was voluntary, and responses were recorded anonymously. Written, informed consent was obtained from each respondent prior to the interview. All relevant permissions were obtained from QECH beforehand, through consultations with key gatekeepers, including administration and staff (36, 37). The study was approved by the National Committee on Research in the Social Sciences and Humanities (NCRSH) of Malawi; Protocol NO. P.03/19/356. Collected data were analysed thematically and coded within the software programme Nvivo, which organises materials and assists with the coding process.

#### 2.3.4. Limitations

Though extensive and useful in understanding the conditions at QECH, the data set would benefit from a comparison with background values. As trash-burning is extensive throughout the city, there were no obvious locations that could be confidently used as representative background values. And although there were several possible areas upwind and outside of town, the logistics and safety involved in accessing them to change the batteries meant that the measured values at Queens could not be compared to a stable background concentration. Knowing the wind direction and velocity would have helped in better identifying the potential sources of the measured PM. However, the necessary equipment was not easily available in Blantyre at the time, and as an exploratory study, the immediate hazards for the staff and patients at QECH are relevant regardless of the burning source.

Knowing the exact burning locations and burning times, especially at the incinerator and the cooking times at the Guardian Shelter (though it is nearly constant), would have helped in better identifying the sources and movements of the plumes. However, given the size of the campus and its 24 h schedule, a much larger research team would have been required to quantify all the burning. Finally, the relative humidity, and the potential for interference was not accounted for; the measurements were not adjusted for humidity which could affect the values, though not enough to significantly alter the key findings and need for immediate action.

#### 2.3.5. Computational reproducibility and data availability

R Statistical Software version 4.2.1, RStudio IDE version 2023.3.0.386, and Quarto scientific publishing system version were used for quantitative data analysis and writing of the manuscript (38–40). A set of additional R packages were used for data wrangling, analysis, and visualisation (41–51).

Raw data and analysis-ready processed data is available as an R package by Schöbitz et al. (52). The data underlying the tables and figures of this manuscript are contained in a repository alongside a reproducible document that contains the analysis code and the written narrative of the manuscript (53).

## 3. Results and discussion

The Malawi Bureau of Standards is the national agency responsible for setting and publishing all standards in the country. At the time of writing, the official website^7^ was not available. However, published work that references Malawian standards (54, 55) indicate that the maximum 24 h PM_10_ value is 25 μg/m^3^ and the maximum annual PM_2.5_ value is 8 μg/m^3^. There is no daily maximum value for PM_2.5_. It should be noted that both of these values are at, or below, the level-4 interim WHO targets.

Therefore, for international comparisons and due to the lack of comprehensive Malawian Standards, we use the WHO recommendations as a basis of comparison for the measured values (55). The relevant particulate matter targets are presented in Table 1.

**Table 1:** Recommended long- and short-term AQG (Air Quality Guidelines) levels and interim targets.

Interim target	1	2	3	4	AQG level
Annual					
PM_2.5_, μg/m^3^	35.0	25.0	15.0	10.0	5.0
PM_10_, μg/m^3^	70.0	50.0	30.0	20.0	15.0
24 h^*^					
PM_2.5_, μg/m^3^	75.0	50.0	37.5	25.0	15.0
PM_10_, μg/m^3^	150.0	100.0	75.0	50.0	45.0

^*^99th percentile (i.e., 3–4 exceedance days per year).

### 3.1. Particulate measurements

#### 3.1.1. Peaks

Due to localised variation (air movement in the immediate area) and the density of the observations, the plotted 5 min data obscures persistent trends. The full set of 5 min data are plotted and presented in Appendix A. However, the number of measured values that exceeded the hazardous limit for both parameters are summarised in Table 2.

**Table 2:** Peaks for both PM_2.5_ and PM_10_ are the number of data points above the WHO interim target 1 (annual) which is the least stringent; the number of observations recorded (n) is provided for reference.

Location	*n*	PM_10_	PM_2.5_
Administration	10,900	171	94
Guardian Shelter	11,032	4,529	5,314
Lighthouse Clinic	11,472	4,365	3,891
Lions Sight	11,749	91	14
Malaria	12,031	617	578
Mercy James	12,193	736	749
Ward 6B	11,861	1,113	1,236
uMoyo	10,637	703	399

Over the course of the 2 month study, the hazardous limits for both parameters were exceeded at all locations except for the Lions Sight hospital. The limits were exceeded fewer than 50 times at five of the locations, and only the monitors at the Lighthouse Clinic and the Guardian Shelter recorded more than 50 instances above the hazardous limits. At the Lighthouse Clinic, the PM_10_ limit was exceeded almost twice as often as the PM_2.5_ limit, while at the Guardian Shelter, the reverse is true. Because the Lighthouse Clinic is within 50 m of the incinerator, the number and predominance of PM_10_ peaks is characteristic of incomplete combustion and dust that is typical for the area. Although there is trash burning at the Guardian Shelter, the main source of smoke is from the constant cooking. The residents at the Guardian Shelter are mostly cooking with coal and wood that is burned within small “improved” cooking stoves that are contained, in a row, within a dedicated cooking block. The fewer number of PM_10_ peaks is a testament to this intervention, though the frequency of PM_2.5_ peaks is still beyond acceptable.

#### 3.1.2. 24 h averages

**Figure 3:**
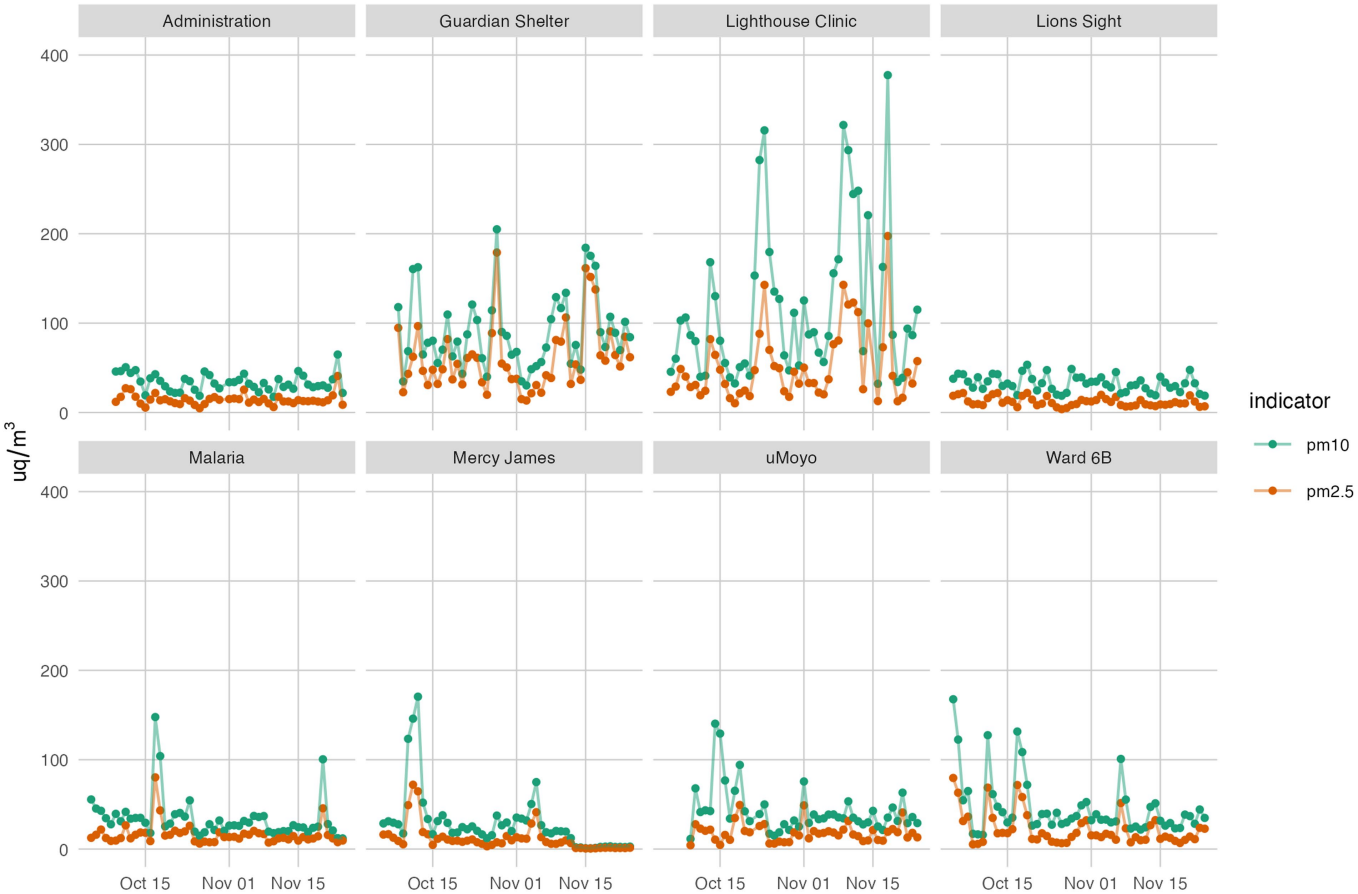
Average 24-hour PM_10_ and PM_2.5_ at 8 monitoring stations over 2 months.

The 24 h averaged values (logged every 5 min) are presented for each location, for both PM_10_ and PM_2.5_ in Figure 3. As well as dampening local variation and peaks, the WHO air quality guidelines are also based on 24 h averages which are the standard against which the health risks can be judged.

Overall, PM_2.5_ values remained below 100 μg/m^3^ at 6 of 8 locations (Administration, Lions Sight, Malaria Project, uMoyo, Ward 6B, and Mercy James); PM_10_ values were consistently below 100 μg/m^3^ at the same locations, but with several average values extending slightly above, and then infrequently.

The daily averages at both the Lighthouse Clinic and the Guardian Shelter are both consistently higher for both parameters and the two averages closely follow the same general trends. Though the peaks at the Lighthouse Clinic were higher than the Guardian Shelter, the low values were consistently lower, indicating more times of little or no burning, unlike the Guardian Shelter emissions which were relatively constant. However, further analysis of 12 h averages (8:00–15:59 (working hours) and 16:00–7:59 (evening)) did not indicate clear differences between the time periods; stated differently, the data did not clearly point to more or less burning in the day or at night.

#### 3.1.3. PM ratios

**Figure 4:**
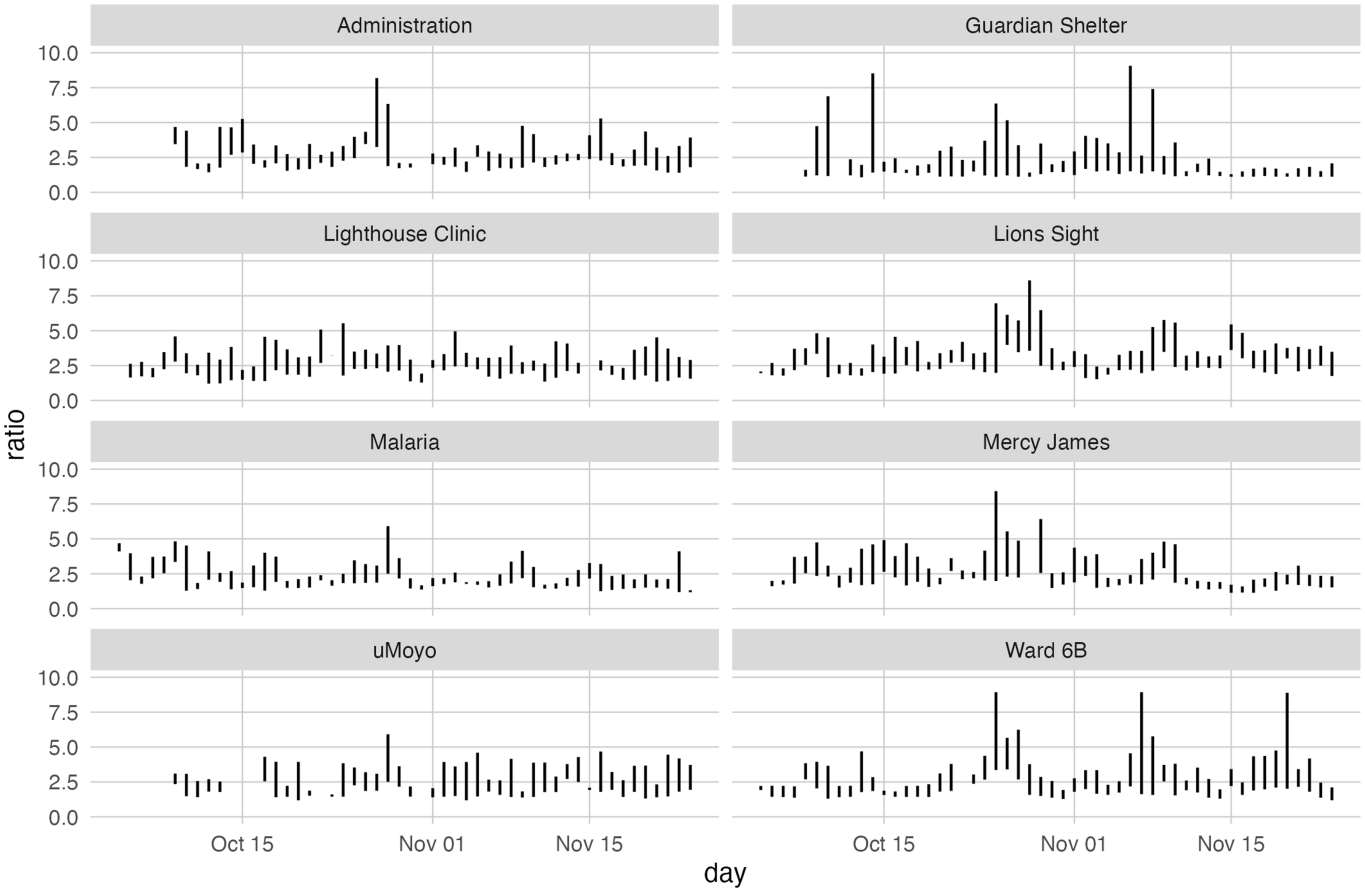
PM_10_:PM_2.5_ values for each location. Each line shows the minimum and maximum ratio for the day based on ratios calculated hourly. Does not include 14th to 16th October at location uMoyo due to extreme outliers.

Given that the type of fuel and the type of burning (contained vs. open) produce very different particulate “fingerprints,” the ratio of PM_10_ to PM_2.5_ values were examined to determine if a clear difference between locations, and therefore source, could be identified. The results are presented in Figure 4.

**Figure 5:**
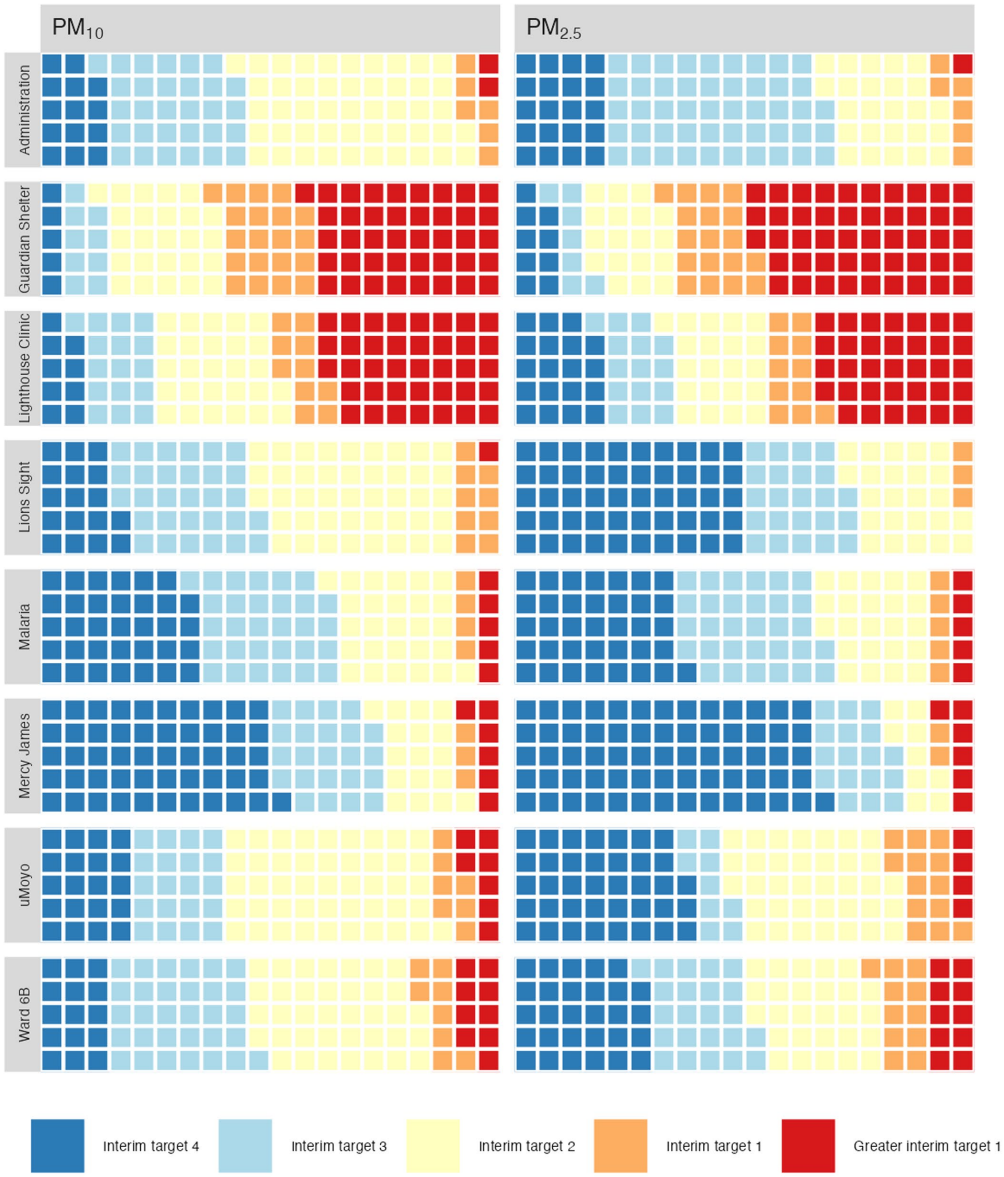
Percentage of measured values categorised according to WHO 2021 targets.

The results presented in Figure 5 illustrate both the presence of PM_10_ relative to PM_2.5_ as well as the range of values over which that ratio is observed.

Unlike the peak values presented in Table 2, the values presented in Figure 5 indicate the relative presence of larger (PM_10_) particles to smaller ones (PM_2.5_) regardless of their concentration: the ratio of two low concentrations can be the same as the ratio of two high concentrations and is therefore more indicative of source than of proximity.

In general, the calculated ratios are concentrated between 1.25 with few values exceeding 7.5. Three from each the Guardian Shelter and the Ward 6B exceeded 7.5 though the most values exceeding 5 were at the Guardian Shelter. It is interesting to note that despite the large daily variations at the Lighthouse Clinic, the parameter ratios there were fairly consistent with few spikes, and only 2 days with ratios above 5.

An examination of the range of ratios is used to better understand the variability within a single day. For example, several of the calculated values at the Guardian Shelter have a range of more than 5, and 1 day had a range of close to 10, i.e., the minimum ratio recorded for that day was close to 1 and the maximum value was over 10. The composition of emissions recorded at that location ranged significantly, and likely reflects a range of burning styles and/or fuel type.

### 3.2. Exposure data

The relative amount of time exposed to a given category of air quality is shown in Figure 5. Each square represents 1% of the measured time.

Unsurprisingly, a majority of the air quality at the Lighthouse Clinic and the Guardian Shelter falls at, or above, interim target 1. What is less expected is that even though there are fewer days of hazardous or very bad air quality at the other locations, most locations do not have target 4 values for even half of the time. Mercy James, the Malaria Project and Lions Sight have the largest percentages of days with air quality meeting the interim target 4, which is expected as these locations are further away from the main burning site. Interim target 2 rated air occupies an unexpectedly large proportion of the days at all locations, indicating that although there are a few very toxic, and a few quite good days, the majority of the air is actually neither, but still cause for concern.

### 3.3. “The air we breathe is not good”: perspectives from QECH

As the previous section has described, the open incineration of waste at QECH has created hazardous conditions for those occupying the space. These risks were not lost on staff and caregivers, as interviews demonstrated broad and near universal awareness of the poor air quality within the hospital grounds. Overwhelmingly, both caregivers and staff were quick to decry the poor quality of the air, generally without prompting. The few exceptions were those staff posted on the peripheries of the hospital grounds, at a distance from the spaces of incineration. However, even those who did not experience the impacts of the burning, were aware of it, and considered themselves fortunate to be posted in a section of the hospital where it was less of a problem. Furthermore, according to caregivers and staff who work night shifts, air quality can be particularly bad at night and in the early morning, due to the habit of janitorial staff concentrating their burning during the late hours. The poor air quality on the hospital grounds is also a frequent cause for complaint by patients and visitors, with nearly every staff member interviewed being able to recall having received a complaint, and in turn, complaining to the administration. One of the staff^8^ members responsible for the burning said that he personally, had received hundreds of complaints, but was powerless to affect meaningful change, aside from burning at different hours, until the incinerator could be repaired.

Despite this consensus that the burning of waste within the hospital was affecting the air quality, significant differences emerged between respondents over their understandings of potential impacts, the effectiveness of various coping mechanisms, and their problematisations linked to the burning of specific waste materials. In addition, interviews revealed that these understandings were informed by a significant amount of misinformation, even amongst trained medical staff, which may contribute to them being less able to mitigate potential risks for themselves and those who rely on their care.

#### 3.3.1. Impacts, problematisations, and misinformation

The poor air quality within QECH was responsible, according to respondents, for a wide array of health impacts. The most common ones cited included: coughing and sneezing, sore throat, stinging eyes, breathing difficulties, and persistent cold and flu. Nausea was also mentioned, but was not a commonly described impact. Only one respondent^9^, of the 26 total interviewed, did not describe lingering health impacts which they could ascribe to the smoke, however, they also described having chronic eye irritation, but did not believe the smoke was a contributing factor.

In addition to these impacts, which respondents bear on a daily basis, many also believed that the smoke could contribute to a number of more serious, long-term health complications. For instance, nearly a quarter of respondents raised concerns of the potential impact that the smoke could have on patients or staff with asthma. Others flagged poor air quality as a potential risk factor for certain cancers, lung disease, or heart problems. For a few, the smoke posed an unknown danger: they were not sure what types of impacts it could have, but they were sure it was harmful in some way.

Also, understandably, given the large tuberculosis ward present within the hospital grounds, and the high prevalence of the disease within Malawi (13) there was significant concern (more than half of respondents) about the impact the poor air quality could have on those with the disease. However, there also persisted a belief among several respondents, including several nurses, that the smoke could be a cause of the disease itself. As one staff member^10^ stated, “I believe breathing this air for a long time can cause tuberculosis.” This, however, was only one of the few instances of misinformation which staff members held regarding air quality and health. Another example, voiced by several respondents, included a belief that some staff members were immune to the impacts of the smoke, because they had received vaccinations from the hospital. Nonetheless, they were concerned about the impacts of the smoke on patients and visitors, as one staff member (footnote 8) expressed:

Personally, I have never experienced [eye discomfort] because I get vaccinated and I am protected including other staff. However, we realise that the air can badly affect other people and patients who come to this place.

Another interesting misconception that emerged, which may be tied partly to translation and transcription, was a different cultural understanding of smoke versus smells. More than half of the respondents appeared to conflate the two, with some expressing a belief that it was the odour of what was being burnt that was harmful, rather than the smoke being given off. This conflation led many to specifically problematise the burning of certain wastes, such as plastics, medicines, and other medical wastes, which give off distinctive or less pleasant odours, as opposed to the burning of other items, such as garden refuse, which may produce significant smoke, and contribute to higher recorded values of particulate matter, but produce a less pungent, or more normalised, odour.

#### 3.3.2. Coping mechanisms

Finally, in order to manage the impacts of QECH’s persistently poor air quality, staff and visitors reported having developed a number of coping mechanisms, designed to help them get through their daily routines. These included staying indoors, blocking doors and windows, and taking breaks away from hospital grounds in order to catch some breaths of fresher air. However, for janitorial staff,^12^ inside was not necessarily better, as several reported that their indoor workspaces were insufferable for long periods of time from the smell of cleaning agents and other chemicals. Furthermore, amongst respondents there was a general disagreement over the effectiveness of personal protective equipment (PPE), such as face masks, towards mitigating the impacts of the smoke. A few staff described pleading to hospital administration for such equipment, but to no avail. However, other staff members, who do have access to PPE, noted that even face masks do little to mitigate the impacts of the smoke, describing them as ineffective.

Most staff have been unable to find any way to mitigate the impacts of the smoke, and only found relief once they reached home at the end of their shift, as one of the janitorial staff described, “we only feel safe when we are home.” Of course, this relief is not available for the hundreds of patients and caregivers who sleep at QECH or are unable to leave. Ultimately, most place their hope in the construction of the new incinerator (which had not yet been activated at the time of the interviews), and biding their time as construction drags on; coping as best they can. As the same member of the janitorial staff (footnote 11) described, “we are just hoping we will start breathing good air soon, when the new incinerator is opened.”

## 4. Conclusion

The air quality, as a result of open waste burning at Queen’s is poor and not suitable for a city, let alone a hospital. Over the course of this 2 month study, the hazardous limits for both PM parameters were exceeded at all locations except for the Lions Sight hospital. The WHO limits were exceeded fewer than 50 times at five of the locations, and only the monitors at the Lighthouse Clinic and the Guardian Shelter recorded more than 50 instances above the most hazardous limit. The results for the Lighthouse Clinic are directly related to the main trash burning site, while the worrisome air quality at the Guardian Shelter is primarily from near constant cooking with charcoal. Simple improvements to the cooking block (e.g., whirlybird vents) could at least vent the smoke above the roof and improve the air quality at human level. The composition of emissions recorded ranged significantly; the ratio PM_10_:PM_2.5_ likely reflects a range of burning styles and/or fuel (waste) type, and no discernible trend was observed. Among interview respondents, there was a general consensus that the air quality caused by the burning of waste within the hospital was a problem, although there were significant differences between respondents over their understandings of potential impacts, the effectiveness of various coping mechanisms, and their problematisations linked to the burning of specific waste materials. For most, going home or leaving the Queen’s campus was the only pathway to relief, though for patients who cannot leave, endurance was the only option.

This work was originally planned as a before-and-after study: we envisioned reporting these baseline results compared to follow-up measurements once the long-promised incinerator was commissioned. However, nearly 4 years later, the incinerator is rarely functional (partly for technical reasons, partly for financial ones) and the smoke hovering over Queen’s is still thick. Waste management has long been known as an environmental health issue, but in the case of open trash burning at a medical facility, it becomes more: for some of the most vulnerable, especially those with tuberculosis, asthma or other chronic disease, it could be a matter of life or death.

## Data availability statement

The datasets presented in this study can be found in online repositories. The names of the repository/repositories and accession number(s) can be found at: https://doi.org/10.5281/ZENODO.7825679.

## Ethics statement

National Committee on Research in the Social Sciences and Humanities (NCRSH) of Malawi; Protocol NO. P.03/19/356. It was conducted in accordance with local legislation and institutional requirements. All participants provided their written informed consent to participate in this study. Written informed consent was obtained from the individual(s) for the publication of any potentially identifiable images or data included in this article.

## Author contributions

ET and MK: conceptualization, methodology, formal analysis, writing – original draft, writing – review & editing, supervision, project administration, funding acquisition. HH: conceptualization, software, and data curation. HC: software, investigation, and data curation. JK: investigation and data curation. LS: software, formal analysis, data curation, and visualization. SV: validation and writing – original draft. All authors contributed to the article and approved the submitted version.

## Footnotes

^1^Sharps are handled and disposed of separately.

^2^There was an older incinerator on the grounds which failed in 2017. Between 2017 and 2021, when the new incinerator was commissioned, nothing was incinerated at all.

^3^Patients are required to bring a caregiver for in-patient hospital stays, usually a family member, who assists with feeding, bathing, and all other non-medical services.

^4^ID5, 2019-11-18.

^5^BMP is credited with the development of the Blantyre Coma Score, which is used widely.

^6^ID44, 2021-01-18.

^7^ www.mbsmw.org

^8^ID3, 2019-11-18.

^9^ID1, 2019-11-18.

^10^ID8, 2019-11-20.

^12^ID14, 2019–1,123.

## Supplementary material

Supplementary material PM10:PM2.5 values for each location. Each line shows the minimum and maximum ratio for the day based on ratios calculated hourly. Does not include 14th to 16th October at location uMoyo due to extreme outliers.
