# Supplementary material for: “It is unbearable to breathe here”: air quality, open incineration, and misinformation in Blantyre, Malawi
Source: Front Public Health. 2023 Oct 12;11:1242726. doi: 10.3389/fpubh.2023.1242726 (PMC10613470; doi:10.3389/fpubh.2023.1242726)
Supplement: Supplementary material — PM10:PM2.5 values for each location. Each line shows the minimum and maximum ratio for the day based on ratios calculated hourly. Does not include 14th to 16th October at location uMoyo due to extreme outliers. [file Table_1.DOCX]

# Appendix A

| Administration | Guardian Shelte | ighthouse Clini | Lions Sight | Malaria | Mercy James | uMoyo | Ward 6B |  |
| --- | --- | --- | --- | --- | --- | --- | --- | --- |
|  | | | | | | |  | pm10 |
|  |  |  |  |  |  |  |  |  |
|  |  |  |  |  |  |  |  |  |
|  |  |  |  |  |  |  |  |  |
|  | | | | | | | |  |

1000

750

500

250

0

uq m^3^

1000

750

500

pm2.5

250

0

Oct 15

Nov 01

Nov 15

Oct 15

Nov 01

Nov 15

Oct 15

Nov 01

Nov 15

Oct 15

Nov 01

Nov 15

Oct 15

Nov 01

Nov 15

Oct 15

Nov 01

Nov 15

Oct 15

Nov 01

Nov 15

Oct 15

Nov 01

Nov 15

Figure 6: PM_2.5_ and PM_10_ values collected every 5 minutes over 10 months at 8 locations.
