# Supplementary material for: “It is unbearable to breathe here”: air quality, open incineration, and misinformation in Blantyre, Malawi
Source: Front Public Health. 2023 Oct 12;11:1242726. doi: 10.3389/fpubh.2023.1242726 (PMC10613470; doi:10.3389/fpubh.2023.1242726)
Supplement: Supplementary file 2 [file Table_2.DOCX]

# Appendix B

*Hardware Components*

1. Raspberry Pi 3 Model B single-board computer technical specifications:
   - Broadcom BCM2837 64bit Quad Core Processor powered Single Board Computer running at 1.2GHz 1GB RAM
   - BCM43438 WiFi on board
   - Bluetooth Low Energy (BLE) on board
   - 40pin extended GPIO
   - 4 x USB 2 ports 4 pole Stereo output and Composite video port
   - Full size HDMI CSI camera port for connecting the Raspberry Pi camera
   - DSI display port for connecting the Raspberry Pi touch screen display
   - Micro SD port for loading your operating system and storing data
   - Upgraded switched Micro USB power source (now supports up to 2.4 Amps)
   - Same form factor as the Pi 2 Model B, however the LEDs have changed position
   - RPI3-MODB-16GB-NOOBS
2. Nova PM SDS011 Sensor
   - See Data Sheet attachment for technical specifications
3. MCP3008 microchip
   - analogue-to-digital converter (ADC)
4. Romoss Sense8+ 30000mAh QC Type-C
   - Power bank battery
5. Pipe
   - clear rubber pipe attainable from any local hardware store
6. Box
   - any box that can fit all the components comfortably can be used. The box will need to have a whole drilled in it for the pipe.

*Software ‘components’ and data management*

1. The Raspberry Pi operating system runs off of Rasbian software on a NOOBS scandisk.
2. Air quality monitoring code is written in Python using the [aqi library](https://pypi.org/project/python-aqi/) (A library of algorithms to convert between AQI value and pollutant concentration). To be able to collect data, the device needs to be connected to a reliable wifi network, the SSID and password need to be coded onto the device during step 2 in the building method.
3. Once the whole device has been put together and placed on site, to access that data we use a combi- nation of programs:
   - [Putty](https://www.putty.org/) - this is an SSH client that we use to gain access to the Raspberry Pi from another computer. To be able to access the Raspberry Pi in question, the computer in use and the air quality monitoring device need to be on the same network for the connection to be successful.
   - [Winscp](https://winscp.net/eng/download.php) - this is an FTP client that we use, in conjunction with Putty, to be able to transfer data files from the air quality monitoring device to one’s computer.
   - [Remote.it](https://app.remote.it/) - this is an application that supports remote SSH, so this is used when wanting to access data from a device that is not connected to the same network as the computer in use. Remote.it is used together with Putty and Winscp to access the air quality data remotely.

*Connection/building method*

To build the sensor, we followed these steps:

1. Set up the Raspberry Pi with the Rasbian software using the NOOBS scandisk. (insert the scandisk into the Raspberry Pi) A comprehensive start up guide can be found here.
2. Next we load the code onto the Raspberry Pi. The code can be found on the Open Data Durban Hos- pital Stations Repository Github. To use it you can follow the steps in the file called Initiate_WS.txt. Once all the steps have been completed, the Raspberry Pi is now a device that can be connected to a particulate matter monitoring sensor.
3. Connect the hardware components
   - Connect the Raspberry Pi to the Nova PM Sensor using the break out board that comes with the Sensor.
   - Connect the battery to the Raspberry Pi via the micro port
   - Attach the rubber pipe to the sensor
   - Assemble all components inside the box, with the pipe coming out of the drilled hole
   - To prevent dirt entering the system, close off all openings between the pipe and the box with silicone putty
   - Secure box in an elevated position (not too close to the ground) and with the pipe facing the direction of the inflow of the air under investigation.
